# Differential Responses of Antioxidative System during the Interaction of Soursop Fruits (*Annona muricata* L.) and *Nectria haematococca* at Postharvest Storage

**DOI:** 10.3390/plants10071432

**Published:** 2021-07-14

**Authors:** Alejandro Rubio-Melgarejo, Rosendo Balois-Morales, Verónica Alhelí Ochoa-Jiménez, Paloma Patricia Casas-Junco, José Orlando Jiménez-Zurita, Pedro Ulises Bautista-Rosales, Guillermo Berumen-Varela

**Affiliations:** 1 Programa de Doctorado en Ciencias Biológico Agropecuarias, Universidad Autónoma de Nayarit, Carretera Tepic-Compostela km. 9, Xalisco C.P. 63780, Nayarit, Mexico; alexelrm1790@hotmail.com (A.R.-M.); balois_uanayar@hotmail.com (R.B.-M.); 2 Unidad de Tecnología de Alimentos-Secretaría de Investigación y Posgrado, Universidad Autónoma de Nayarit, Ciudad de la Cultura S/N, Colonia Centro, Tepic C.P. 63000, Nayarit, Mexico; veronica.ochoa@uan.edu.mx (V.A.O.-J.); mcapalomaa@outlook.es (P.P.C.-J.); zurit_8@hotmail.com (J.O.J.-Z.)

**Keywords:** antioxidant activity, early response, total soluble phenols, *polyphenol oxidase*, *superoxide dismutase*, gene expression

## Abstract

Soursop fruit (*Annona muricata* L.) production is diminished by the attack of pathogens such as *Nectria haematococca*. However, the fruit–pathogen interaction at the biochemical and molecular levels is still unknown. The objective of this study was to analyze the response of the soursop fruit to the presence of *N. haematococca* during postharvest storage. Soursop fruits were inoculated with the pathogen and total phenolic compounds, antioxidant capacity by Ferric reducing/antioxidant power (FRAP), 2,2′-azinobis-(3-ethylbenzothiazoline-6-sulfonate) (ABTS•+), and 2,2′-diphenyl-1-picrylhydrazyl radical (DPPH•), as well as enzymatic activity and transcript levels of *polyphenol oxidase (PPO)* and *superoxide dismutase (SOD),* were evaluated at 1, 3, and 5 days of storage. The noninoculated fruits were the controls of the experiment. The highest total phenol content was recorded on day one in the inoculated fruits. FRAP, ABTS, and DPPH activity presented the highest values on day three in the control fruits. Inoculated fruits recorded the highest *PPO* activity on day five and a five-fold induction in the *PPO* transcript on day three. *SOD* activity showed a decrease during the days of storage and 10-fold induction of *SOD* transcript on day three in the inoculated fruits. Principal component analysis showed that total phenols were the variable that contributed the most to the observed variations. Furthermore, a positive correlation between total phenols and *SOD* activity, *PPO* expression, and *SOD* expression, as well as between DPPH and FRAP, was recorded. The results showed a differential response in antioxidant capacity, enzymatic activity, and gene expression during the interaction of soursop fruits–*N. haematococca* at postharvest storage.

## 1. Introduction

Mexico is the largest worldwide producer of soursop (*Annona muricata* L.), and Nayarit is the top leader producer of this fruit [1]. It is a climacteric fruit that presents a high respiration rate and is highly perishable at ambient temperature [2,3]. The development and ripening of climacteric fruits are oxidative processes, producing reactive oxygen species (ROS) such as superoxide radical (O_2_^−^) and hydrogen peroxide (H_2_O_2_) [4]. On the other hand, one of the main problems of the soursop fruit is the attack by fungi, which prevents its commercialization to national and international markets. Soursop fruits are infected by various species of fungi, such as *Aspergillus flavus, Aspergillus niger, Botryodiplodia theobromae, Colletotrichum sp., Fusarium solani, Mucor sp., Penicillium chrysogenium, Penicillium sp., and Rhizopus stolonifera* [3]. *N. haematococca* (anamorph: *F. solani*) is a pathogen that is located in the soil and infects plants by emitting a germ tube, which crosses the root tissues and colonizes the xylem vessels until they obstruct them and prevent translocation of water and nutrients to other organs of the plant [5]. Nevertheless, this pathogen has also been reported as one of the main pathogens that cause rot in the soursop fruit [6]. Given the multiplicity of interactions, plants present a wide flexibility of responses to beneficial organisms and pathogens through activated signals and adaptive responses of the plant to pathogens, generating a sophisticated network of signals; the processes of synergism and antagonism between pathways of signaling allows to refine the most adequate defense mechanism [7]. Rubio-Melgarejo et al. [8] mentioned that the production of secondary metabolites such as phenols, and the increase in oxidative levels, play an essential role in interactions such as chemical defense against pathogens, inhibiting their development. One of the plant defense mechanisms is the enzymatic antioxidant system, as well as the regulation and production of reactive oxygen species (ROS) for the defense in plants [9]. The accumulation of phenolic compounds has been related to disease resistance in plant–pathogen interactions [10]. 

Chandrasekaran and Chun [11] mention that oxidative enzymes such *as polyphenol oxidase (PPO)* catalyze the oxygen-dependent oxidation of phenols to quinones (antimicrobial compounds that are implicated in the lignification of plant cell wall during the microbial invasion) involved in plant defense. In turn, *superoxide dismutase (SOD)* is the first essential antioxidative enzyme that prevents damage caused by oxidative stress [4]. These enzymes may be involved in response to the defense reaction and hypersensitivity against fungal infection. However, few studies exist at the biochemical and molecular level of the antioxidant system in tropical and subtropical fruits. Disease resistance is related to the activation of defense responses at the biochemical and molecular levels that activate at certain stages during the plant–pathogen interaction. Taking this into account, it requires comprehensive studies to understand the response mechanisms to pathogen infection. Nevertheless, there are no scientific investigations that have reported and evaluated the soursop fruit–*N. haematococca* interaction at a biochemical and molecular level. Therefore, the objective of the study was to evaluate the response of the antioxidant, enzymatic, and gene expression of soursop fruits to the presence of the pathogen *N. haematococca* during postharvest.

## 2. Results

### 2.1. Total Phenolic Compounds

**Figure 1:**
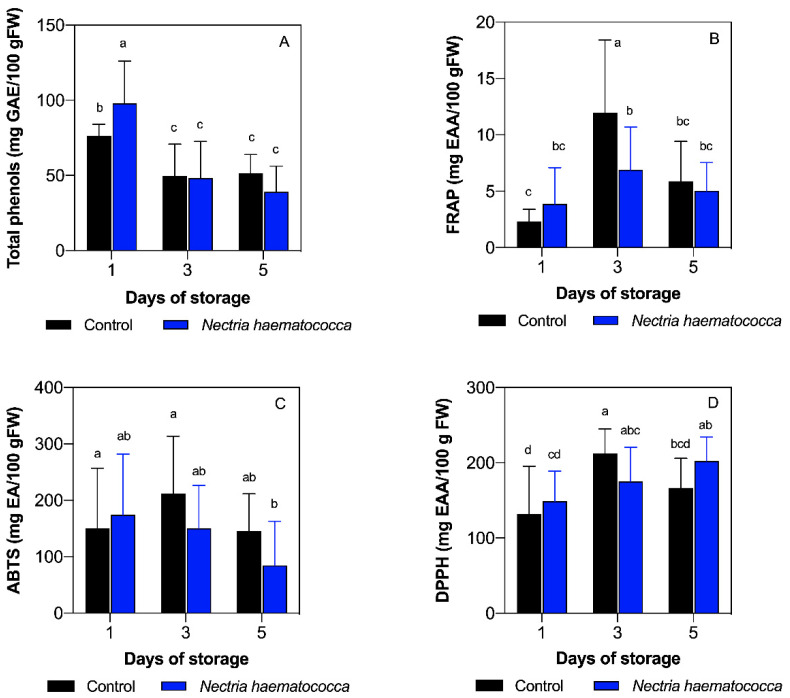
Content of total phenols (**A**), antioxidant capacity by the ferric reducing/antioxidant power (FRAP) (**B**), 2,2′-azinobis-(3-ethylbenzothiazoline-6-sulfonate) (ABTS•+) (**C**), and 2,2′-diphenyl-1-picrylhydrazyl radical (DPPH•) (**D**). Different letters indicate significant differences according to Tukey’s multiple comparison test (*p <* 0.05). Highest mean values are described with the letter a. Vertical lines represent the standard deviation of the means.

The fruits inoculated with *N. haematococca* presented the highest concentration of total phenols (97.98 mg EAG/100 g.f.w.) on day one, compared to all the conditions analyzed according to Tukey’s multiple comparison test (*p* < 0.05), as observed in Figure 1A. On the other hand, a significant decrease was recorded in fruits inoculated with *N. haematococca* on day three (48.27 mg EAG/100 g.f.w.) and day five (39.04 mg EAG/100 g.f.w.), compared to day one (*p* < 0.05), as shown in Figure 1A. No differences were exhibited between inoculated and control fruits on these days (*p* > 0.05) 

### 2.2. Ferric Reducing/Antioxidant Power (FRAP) Capacity

The highest FRAP capacity was displayed in control fruits at day three (11.96 mg EAA/100 g.f.w.) compared to the rest of the conditions tested (*p* < 0.05). Moreover, inoculated and control fruits significantly increased their antioxidant capacity at day three in comparison to control fruits on day one (*p* > 0.05). Finally, inoculated and noninoculated fruits decreased their antiradical capacity on day five regarding control fruits at day three, as shown in Figure 1B. Furthermore, an increasing tendency from day one to three followed by a decrease on day five was observed in control fruits (Figure 1B). 

### 2.3. 2,2′-Azinobis-(3-ethylbenzothiazoline-6-sulfonate) (ABTS•+) Antioxidant Capacity

The antioxidant capacity evaluated by ABTS•+ showed the highest value at day three of storage in control fruits (211.97 mg EAA/100 g.f.w.) and the lowest value at day five in fruits inoculated with *N. haematococca*. Similar values were found at day one in inoculated (174.29 mg EAA/100 g.f.w) and control fruits (150.21 mg EAA/100 g.f.w.) (*p* > 0.05). Furthermore, only control fruits at day three showed significant differences compared to day five in inoculated fruits, showing a dramatic decrease among them (*p* < 0.05), as shown in Figure 1C.

### 2.4. 2,2′-Diphenyl-1-picrylhydrazyl Radical (DPPH•) Antioxidant Capacity 

The antiradical capacity evaluated by the DPPH• showed no significant differences between fruits inoculated (148.86 mg EAA/100 g.f.w.) and control (131.87 mg EAA/100 g.f.w.) on day one (*p* > 0.05). On the other hand, fruits inoculated with *N. haematococca* showed values of 208.02 mg EAA/100 g.f.w. on the third day of storage, showing higher values than the control fruits on day one, and no differences were reported when compared to control fruits on day three with values of 212.14 mg (*p* > 0.05). Finally, on the fifth day of storage, the antioxidant capacity of the control fruits decreased (166.64 mg EAA/100 g.f.w.) (Figure 1D). Additionally, fruits inoculated with *N. haematococca* presented a gradual increase according to the days of storage, as shown in Figure 1D.

### 2.5. Polyphenol Oxidase (PPO) and Superoxide Dismutase (SOD) Enzyme Activity

**Figure 2:**
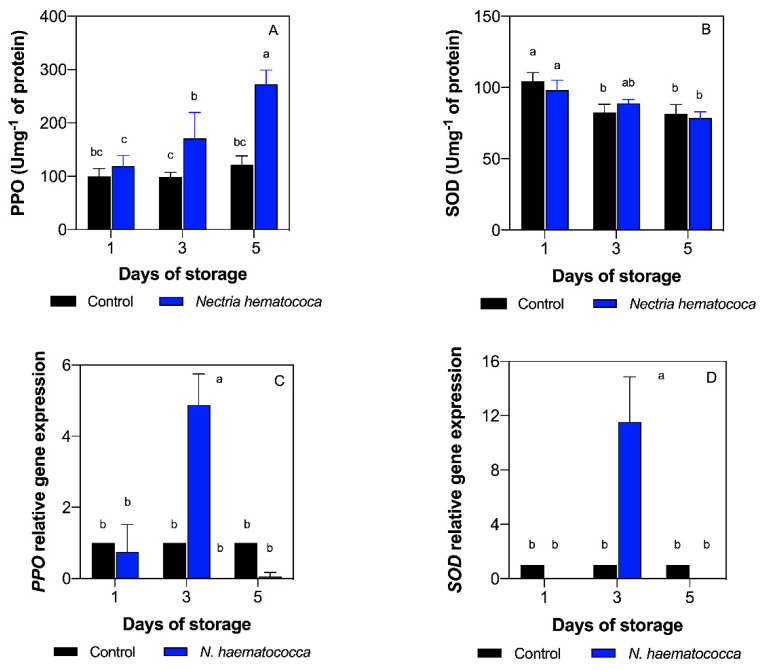
Enzymatic activity of *polyphenol oxidase* (*PPO*) (**A**) and *superoxide dismutase* (*SOD*) (**B**), as well as relative gene expression of *PPO* (**C**) and *SOD* (**D**) by quantitative real-time polymerase chain reaction (qRT-PCR) in soursop fruits at postharvest storage. Different letters indicate significant differences according to Tukey’s multiple comparison test (*p <* 0.05). Highest mean values are described with the letter a. Vertical lines represent the standard deviation of the means.

*PPO* activity exponentially increased during postharvest storage in the fruits inoculated with *N. haematococca* (*p <* 0.05). Indeed, fruits inoculated with *N. haematococca* showed an increase of *PPO* activity (171 Umg^−1^ of protein) on day three compared to the control (*p <* 0.05), as shown in Figure 2A. On the other hand, control fruits showed similar values during postharvest storage, presenting the lowest *PPO* activity on day three (98 Umg^−1^ of protein). Furthermore, fruits inoculated with *N. haematococca* substantially increased on day five (121 Umg^−1^ of protein), reaching the maximum *PPO* activity (272 Umg^−1^ of protein) under all the conditions tested (*p <* 0.05), as can be seen in Figure 2A. In addition, positive interaction between days of storage and inoculation was found in *PPO* activity (Table S1), demonstrating that postharvest storage and infection by the pathogen have a combined effect on *PPO* activity. 

On the other hand, *SOD* activity decreased during postharvest storage in control and inoculated fruits. However, no significant differences were recorded between control and inoculated fruits when they were compared for days of storage (*p* > 0.05).

### 2.6. Relative Gene Expression (PPO and SOD)

To gain further insight into the oxidative reaction in the fruit–pathogen interaction during postharvest storage, the relative gene expression of the *PPO* and *SOD* genes were analyzed by quantitative real-time polymerase chain reaction (qRT-PCR), as shown in Figure 2C,D. The highest transcript level of *PPO* was recorded on day three of storage in the fruits inoculated with *N. haematococca*, showing a five-fold increase compared with the control fruits (*p <* 0.05), as shown in Figure 2C. On the other hand, a 10-fold increase of *SOD* transcript levels was recorded on the third day of storage, presenting the highest value between all the conditions evaluated (*p <* 0.05), as observed in Figure 2D. *PPO* and *SOD* transcript levels showed no differences in the other days evaluated in comparison with the control (*p >* 0.05). *PPO* and *SOD* transcript levels showed positive interaction between day of storage and inoculation (Table S1), demonstrating that postharvest storage and infection by the pathogen have a combined effect on both transcripts.

### 2.7. Multivariate Analysis

**Figure 3:**
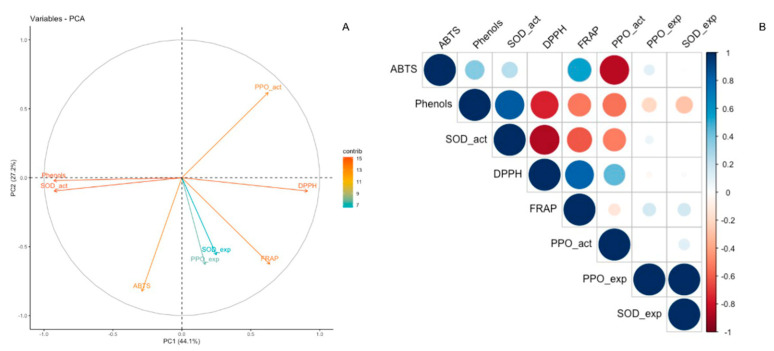
Principal component analysis (PCA) biplot (**A**) and correlation plot of the variables (**B**). The color indicates the contribution or the correlation of the variables.

Principal component analysis (PCA) and correlation analysis were applied to observe the relationship among variables under the conditions evaluated (Figure 3A,B). PCA biplot showed that two dimensions explain 71.3% of the variations. About 44.1% of the variation in the treatments was explained by PC 1, showing that total phenols and *SOD* activity were the variables that contributed the most (Table S2). On the other hand, 27.2% of the variation was explained by PC 2 (Figure 3A), where the variables that most contributed to PC 2 were ABTS and *PPO* activity. The PCA results indicate that total phenols contributed the most to the observed variations, whereas *SOD* expression had the least effect on fruit–pathogen interaction during postharvest storage (Figure 3A). This result is corroborated by the significantly positive correlation between total phenols and *SOD* activity (Figure 3B). Furthermore, pairwise Pearson analysis demonstrated high correlations between *PPO* expression and *SOD* expression, as well as between DPPH and FRAP (Figure 3B). This analysis also showed that ABTS presented a positive correlation with phenols, *SOD* activity, FRAP, and *PPO* expression (Tables S3 and S4).

## 3. Discussion

Previous studies have identified that *N. haematococca* is one of the main pathogens that attack soursop fruit [6]. However, these authors did not evaluate the mechanism of fruit–pathogen interaction during postharvest storage. *N. haematococca* was able to increase the levels of phenolic compounds in soursop fruits on day one compared to the control fruits (Figure 1A). This may be due to the induction of the production of phenolic compounds as biochemical inhibitors in response to the presence of the fungus. Several authors [12,13,14] mention that among the first reactions generated by the plant in response to the presence of fungi is the production of phytoalexins (phenolic compounds), toxic compounds for a wide spectrum of fungi and bacteria. This type of response by the host is part of the systemic resistance to diseases in plants and is also known as an early response [12]. Phenols have been shown to have an important role in the defense of plants against different biotic and abiotic factors [15]. On the other hand, the decrease in the concentration of phenolic compounds that occurred on days three and five in fruits inoculated with *N. haematococca* may be because certain fungi and bacteria can modify their functions by secreting effectors within the host that alter genes related to defense [16]. 

On the other hand, phenolic compounds, together with some nitrogenous compounds and vitamins, determine the antioxidant capacity, which can be determined by multiple reactions. This is why it is necessary to evaluate by different methods, such as FRAP, ABTS•+, and DPPH• [17]. In this regard, FRAP and ABTS methods showed a similar trend on days one and three. 

Antioxidant activity can depend on genetic factors, edaphoclimatic conditions, varieties, and biotic stress [18]. The antioxidant capacity of iron reduction (FRAP) is related to the high metabolic activity of the soursop fruit, coupled with the decrease or increase of phenolic compounds [2]. These compounds are involved in the response of plants against beneficial organisms and pathogens with superposition of some active signals by the plant and adaptive responses that help to protect against aggressive pathogens [7]. Rubio-Melgarejo et al. [8] reported, in soursop fruits non-inoculated and inoculated with *Colletotrichum gloeosporioides,* a trend of increase from day one to day five of storage in the FRAP activity. In this study, control fruits showed an increase in their FRAP capacity from day one to three, showing similar results to those previously mentioned.

In fruits inoculated with the pathogen, there were no differences in the FRAP capacity during the days of storage. This can be due to *Fusarium* species producing phytotoxins such as enniatins and fusaric acid, which increase virulence of the fungus and are toxic to the plant [13,19]. 

In the case of the antioxidant capacity ABTS•+, Márquez-Cardozo et al. [2] mention that the increase in the antiradical capacity ABTS•+ may be due to the high metabolic activity of the soursop fruit. This explains the behavior of the antioxidant capacity on the ABTS•+ radical of the control fruits on day three of storage and may be due to the high metabolic activity typical of ripening and the climacteric period after harvest. In turn, Spoel and Dong [20] mention that the increase in ABTS•+ could be to an adaptation mechanism of plants, in their ability to recognize and respond quickly to a possible invader through defense responses such as the production of secondary metabolites such as phenolic compounds. Furthermore, Rubio-Melgarejo et al. [8] reported an increase in antioxidant capacity on the radical ABTS•+ on day one in soursop fruits inoculated with *C. siamense*. Our results showed a decrease in the antioxidant capacity in inoculated fruits, suggesting that it can alter the signal network and manipulate the defenses using virulence effectors. Furthermore, this trend could be associated with the significant decrease of phenolic compounds due to the positive correlation between ABTS and phenols. 

The DPPH• method has the ability to trap the DPPH• radical, which could be linked to some phenolic compounds in the soursop fruit that are capable of neutralizing free radicals. Thus, the antioxidant capacity DPPH• of the soursop fruit may be due to the presence of the hydroxyl groups of phenolic compounds [21]. Gómez and Rodríguez [22] mention that this type of antiradical capacity may be related to the resistance induced in plants due to the presence of pathogenic and beneficial organisms. On day three of storage, an increase in the antioxidant capacity of DPPH• was observed in inoculated and control fruits. The increase in antioxidant capacity on day three may be due to an increase in metabolism as the climacteric peak approaches. On the other hand, the fruits inoculated with the pathogen had a lower antioxidant capacity of DPPH• which could be related to the production of phytotoxins by the fungus and it could increase its pathogenicity. The decrease in the antiradical DPPH• capacity of the control fruits, in comparison to the fruits inoculated with the pathogen on day five, may be related to the fact that the fruits reached senescence and are part of the normal metabolism of the soursop fruit ripening as reported by Balois-Morales et al. [23].

Otherwise, control fruits maintained constant *PPO* activity. Studies carried out by Lima et al. [24] indicate that during the soursop fruit ripening, the enzymatic activity of *PPO* increased from the first to the fourth day of evaluation. Jiménez-Zurita et al. [25] mention that the activity of the *PPO* increased in senescence soursop fruits. It has been reported that, in immature olive fruits (*Olea europea* L.), *PPO* is attached to the chloroplast; however, in ripe fruits, the *PPO* is essentially soluble. The findings of this research suggest that the fruits did not reach the senescence, which explains why the control fruits showed no increase in the *PPO* activity. 

We found that *N. haematococca* dramatically increased the *PPO* activity during ripening. The fruits inoculated with the pathogen showed the highest *PPO* activity on day five. A possible explanation is that the enzyme is located exclusively in plastids and is released into the cytosol after damage, senescence, or deterioration of the organ [26], which would explain the considerable increase in *PPO* activity in fruits inoculated by *N. haematococca* compared to the control, due to the fact that the pathogen caused damage or deterioration in the soursop fruits. When the pathogen is able to overcome the host’s protective responses, it leads to localized necrosis in the fruits [27]. Moreover, the fruit recognizes specific elicitors of the pathogen and activates the defense mechanisms that lead to the hypersensitive response (HR) in the fruit. Many fruits have an enzymatic system capable of using phenolic compounds as a substrate, within which is the *PPO* enzyme, related to the pulp-darkening processes [28]. Therefore, the biochemical inhibitors produced in response to the damage by the pathogen are associated with the production of compounds that, if present in high concentrations, will inhibit the development of the pathogen. The activity of the *PPO* enzyme is higher in the tissues of resistant varieties than those that are susceptible. The importance of *PPO* as a defense mechanism is because it catalyzes the oxidation of phenolic compounds to produce highly reactive quinones, which are often more toxic than the original phenols [29]. These would explain the behavior of the enzymatic activity in the different conditions; this means that when there is a high concentration of phenolic compounds, the *PPO* activity is low and vice versa, which coincides with the negative correlation found between these two variables. Hence, resistance or susceptibility depends on the time it takes for the host to recognize the pathogen and how quickly it activates its defense mechanisms. Furthermore, *PPO* gene expression was also evaluated, finding that *N. haematococca* induced the gene expression at day three in soursop fruits. This suggests that the pathogen triggers molecular defense mechanisms at the maturity of consumption (day three). Strong induction of *PPO* has been reported in leaves of potato plants 24 h after wounding [30]. Moreover, *PPO* gene expression was induced by *Xanthomonas arboricola* pv. ju-glandis 417 in walnut Serr cultivar leaves at 24 h and 72 h [31]. These reports agree with the findings of this experiment. Even when high *PPO* activity and *PPO* gene expression at day three was observed, different behavior and no correlation was found between these two response variables. One possible explanation is that the *PPO* gene family possesses multiple members with distinct biological roles, which causes differential expression patterns.

On the other hand, it has been reported that during the maturation process large amounts of superoxide (O^2−^) are produced, particularly in the deterioration of the membranes, since it can cause the oxidation of the unsaturated fatty acids present in the phospholipids of the membrane [4]. One of the mechanisms to protect the fruit from oxidative stress is through the production and activity of antioxidant enzymes such as *superoxide dismutase* (*SOD*), which has great importance in the antioxidative process [32]. The decrease in *SOD* activity may be related to decreased production of reactive oxygen species as maturation progresses. Regarding the fruits inoculated with *N. haematococca*, Villa-Martínez et al. [13] mention that one of the main reactions that plants generate in response to the presence of pathogens is the production of reactive oxygen species. The fruits can tolerate pathogens through physical and chemical barriers, or through induced defenses that are activated once the host detects the presence of the pathogen, triggering the oxidative explosion during the first hours of the interaction. This reaction leads to the generation of ROS such as superoxide (O^2−^) and or hydrogen peroxide (H_2_O_2_) [27]. In turn, pathogens also produce reactive oxygen species to increase their virulence [33]. The accumulation of reactive oxygen species in plants, both those produced by themselves and by the pathogen, can cause damage to the plant tissue at the molecular level and, ultimately, cause its death [33]. The enzyme *superoxide dismutase* acts as a cellular defense against ROS [34]. Nevertheless, in this study, no association was found between *SOD* activity and the presence of the pathogen. It is known that there must be a high oxidative metabolism with high consumption of O^2−^. 

Comparing the behavior of *PPO* and *SOD* activity, a negative correlation was found between the two enzymes studied, since although both are part of the defense mechanism in plants, their modes of action are different, since *polyphenol oxidase* catalyzes the oxidation of phenols to quinones, and *superoxide dismutase* is an antioxidant by trapping reactive oxygen species [4]. As previously mentioned, we also evaluated the *SOD* gene expression at different days of storage. We found that *SOD* transcript levels had a similar trend compared to the *PPO* transcript levels, finding the highest expression at day three of storage in fruits inoculated with *N. haematococca.* On the other hand, different behavior and no correlation was found between *SOD* activity and *SOD* gene expression. This is similar to the results obtained in the *PPO* analysis, suggesting the same as previously mentioned in the *PPO* analysis, that different members of the *SOD* gene family are regulated depending on the tissue, having distinct biological roles, which causes differential expression patterns. Huan et al. [35] analyzed the gene expression of several *SOD* antioxidant genes in peach fruits, finding different expression patterns depending on the *SOD* gene and stage of development. On the other hand, Lightfoot et al. [36] analyzed the *superoxide dismutase* gene expression (HvCSD1) by a semiquantitative reverse transcription-polymerase chain reaction in the barley–*Pyrenophora teres* f. *teres* (Ptt) interaction. The results of that investigation showed that the HvCSD1 gene was induced at 48 and 72 h postinoculation in sloop and resistant barley breeding line CI9214. Furthermore, a *superoxide dismutase* (OY-*sodA*) gene from plant pathogenic phytoplasma was evaluated in plant and insect hosts by qRT-PCR, showing similar expression levels in both systems [37]. Moreover, high correlation between *PPO* and *SOD* transcript levels was recorded in this study. Altogether, these results suggest that *PPO and SOD* genes are induced by *N. haematococca* to colonize the host. On the other hand, Zhang et al. [38] applied ozone to cantaloupe melon fruits, evaluating the expression profiles of eight *SOD* genes using qRT-PCR. The results of that investigation showed that the transcription level of Cu/Zn-*SOD*-1 gene was the highest in pericarp and pulp of cantaloupe melons treated with ozone, as well as controls. As can be seen, few reports can be found regarding the gene expression in the Annonaceae family. In this context, Prieto et al. [39] analyzed the mRNA levels of a *PPO* gene in chirimoya by qRT-PCR in different organs, showing a high amount of transcript in leaves and flowers. In this study, high expression was found in fruits inoculated with the pathogen at day three of storage in the *PPO* and *SOD* gene. 

## 4. Materials and Methods

### 4.1. Plant Material

Soursop fruits were harvested at 175 days after anthesis from a commercial orchard located in Lima de Abajo, Compostela, Nayarit (21°05′ N; −105°11′ W; 30 m.a.s.l.), reaching the physiological maturity according to [3]. The fruits were immediately transferred to the laboratory, selecting 54 fruits according to peel color, shape, size, and without mechanical, physical, and pathogenic damage. Afterwards, the fruits were disinfected by immersion with sodium hypochlorite at 1.5% for two min and then rinsed with distilled water. Furthermore, 18 fruits per day of storage were used for the subsequent experiments (nine fruits inoculated with *N. haematococca* and nine noninoculated fruits). 

### 4.2. Inoculation of Fruits

The pathogen *N. haematococca* was previously isolated and identified by [6] from soursop fruits. Soursop fruits were inoculated by immersion for one min with a spore suspension of *N. haematococca* at 1 × 10^5^ spores/mL according to the methodology described by Bautista-Rosales et al. [40]. The fruits were stored at 28 ± 2 °C with a relative humidity of 95%. The fruits were evaluated at 1, 3, and 5 days of storage.

### 4.3. Analysis of Total Extractable Phenolic Compounds and Antioxidant Capacity

#### 4.3.1. Crude Extract 

A crude extract was obtained according to the protocol reported by Jiménez-Zurita et al. [25]. In this context, 1 g of mesocarp was homogenized from three soursop fruits for each of the conditions analyzed with distilled water using an Ultraturrax (T8 IKA^®^, Staufen, Germany). Subsequently, it was centrifuged (Z326K Hermle, Wehingen, Germany) at 10,410 *g* for 15 min at 4 °C. The supernatant was recovered and used to determine the absorbance in sextuplicate of the total phenolic compounds and antioxidant capacity.

#### 4.3.2. Total Extractable Phenols

The content of total extractable phenols was determined with the method described by Singleton et al. [41]. A calibration curve was made with gallic acid (Sigma-Aldrich, Shanghai, China) (0–400 mg L^−1^) and the determinations were carried out at 760 nm in a spectrophotometer (Biotek Synergy HT, Hampton, NH, USA). The results were expressed in equivalent mg of gallic acid per gram of fresh weight (mg EGA/100 g.f.w)

#### 4.3.3. Antioxidant Capacity by the DPPH Method

The antioxidant capacity by the DPPH• was performed with the method of Brand-Williams et al. [42]. The change in absorbance at 517 nm was measured in a spectrophotometer (Biotek Synergy HT, USA). Antioxidant capacity was determined using a calibration curve with ascorbic acid (0–100 mg L^−1^). The results were expressed in mg equivalents of ascorbic acid (mg EAA/100 g.f.w).

#### 4.3.4. Antioxidant Capacity by the ABTS•+ Method

Antioxidant capacity by the ABTS•+ method was carried out by the method described by Re et al. [43]. The absorbance was measured at 734 nm in a spectrophotometer (Biotek Synergy HT, USA). Antioxidant capacity was determined using a calibration curve with ascorbic acid (0–150 mg L^−1^). The results were expressed in mg equivalent of ascorbic acid (mg EAA/100 g.f.w).

#### 4.3.5. Iron Reduction Antioxidant Capacity (FRAP)

The iron-reducing antioxidant capacity (FRAP) was measured by the method described by Yen et al. [44]. The absorbance was quantified at 700 nm using a spectrophotometer (Biotek Synergy HT, USA). The reducing capacity was determined with a calibration curve with ascorbic acid (0–30 mg L^−1^). The results were expressed in mg ascorbic acid equivalents (mg EAA/100 g.f.w).

### 4.4. Enzymatic Activity

#### 4.4.1. Crude Enzyme Extraction

A crude enzyme extract to measure the enzymatic activity of *PPO* and *SOD* was obtained. In this regard, 0.5 g of mesocarp from three soursop fruits were independently homogenized with 3.5 mL of 100 mM Tris-HCl (pH 7.1) cold buffer containing 1% polyvinyl pyrrolidone (PVP) and 1 M phosphate (pH 7.8) cold buffer for the determination of *PPO* and *SOD*, respectively. After that, they were individually mixed with an Ultraturrax tissue homogenizer (T8 IKA^®^, Staufen, Germany) and then centrifuged (Z326K Hermle, Wehingen, Germany) at 10,410× *g* for 20 min at 4 °C [25]. The quantification of the enzymatic activities was performed in triplicate.

#### 4.4.2. *Polyphenol Oxidase* (EC. 1.14.18.1; *PPO*)

The enzymatic activity was determined with the methodology described by Lamikanra [45]. In this regard, 3 mL of 60 mM catechol dissolved in a 100 mM Tris-HCl buffer (pH 7.1) and 200 µL of the enzyme extract were used. The analyses were carried out at a temperature of 24 ± 0032 °C. The change in absorbance at 420 nm was measured in the spectrophotometer (UV-5100). Enzyme activity was reported in international units per gram of protein (U mg^−1^ of protein). One unit of enzyme activity is equal to the formation of 1 μmol of o-benzoquinone min^−1^. The soluble protein was determined by Bradford [46] using the enzyme extract of *PPO*.

#### 4.4.3. *Superoxide Dismutase* (EC. 1.15.1.1; *SOD*)

*SOD* activity was evaluated using the method of Beyer and Fridovich [47]. The following reaction mixture was used: 66 mL of a 0.1 M phosphate buffer solution, pH 7.8 (0.01 mM EDTA, 3.66 mL of L-methionine, 2.44 mL of nitro blue tetrazolium, and 1.83 mL of Triton X-100). Next, 500 µL of enzyme extract and 30 µL of riboflavin were added to 3 mL of this reaction mixture. After this, the reaction mixture was illuminated for 7 min with a 20-watt Grolux fluorescent light lamp and then the absorbance was read. The analyses were carried out at a temperature between 24 ± 2 °C. The absorbance was measured in a spectrophotometer (UV-5100) at 560 nm. The enzymatic activity was presented as U mg^−1^ of protein, and each unit of *SOD* was equal to the amount of photoinhibition of the formation of 50% of nitro tetrazolium formazan blue. The soluble protein was determined by Bradford [46] using the enzyme extract of *SOD*.

### 4.5. Relative Gene Expression 

#### 4.5.1. RNA Extraction and cDNA Synthesis

Total RNA was extracted from 0.075 g of frozen pulverized mesocarp tissue at 1, 3, and 5 days of storage using the Spectrum Plant Total RNA kit (Sigma) following the manufacturer’s instructions. Total RNA was quantified in a spectrophotometer (Synergy HT/Take3, BioTek Instrument Inc., Winooski, VT, USA). The integrity of RNA was visually analyzed on a 1.5% agarose gel using a Benchtop UV transilluminator and PhotoDoc-It system for image capture (Laboratory Equipment, Hayward, CA, USA). RNA with a 260/280 ratio between 1.8 to 21 were used for the synthesis of cDNA. The cDNA libraries were generated using the SuperScript III kit according to the manufacturer’s instructions. The cDNA was stored at −20 °C until use.

#### 4.5.2. Gene Selection and Primer Design

Bioinformatics analysis was carried out to identify *PPO* and *SOD* genes in soursop according to the method described by Berumen-Varela et al. [48]. In this regard, a BLAST database was built from a soursop leaf transcriptome (http://www.onekp.com/public_data.html, accessed on 16 June 2021) and then a BLAST search was performed using *PPO* and *SOD* sequences from other species to identify homologous genes. Sequences with 80% or more similarity were selected for primers design. Primers were designed using the Primer Quest Tool (Integrated DNA Technologies, Coralville, IA, USA) which are shown in Table 1. The specificity of the primers was tested by the Primer-Blast tool (http://www.ncbi.nlm.nih.gov/tools/primer-blast/, accessed on 16 June 2021).

#### 4.5.3. PCR

*SOD* and *PPO* transcripts were amplified with PCR in a T-100 thermal cycler (Bio-Rad Laboratories, Inc., Hercules, CA, USA) using the RedTaq Ready Mix (Sigma) following the manufacturer’s instructions. The PCR conditions were initial denaturation at 94 °C for 5 min, followed by 38 cycles of 95 °C for 30 s, 56 °C for 35 s, and 72 °C for 40 s, and a final extension of 72 °C for 10 min. Amplicons were analyzed by 1.5% agarose gel and visualized in a PhotoDoc-It Imaging System (Ultra-Violet Products, Ltd., Cambrige, MA, USA). The PCR products were sequenced to confirm their identity at Macrogen Inc. (Seoul, Korea).

#### 4.5.4. Gene Expression Analysis by qRT-PCR

Relative standard curves were made using four-fold serial dilutions of cDNA for each condition analyzed. The qRT-PCR was performed in a qTOWER3 G touch thermal cycler (Analytik Jena) in real time using the using Maxima SYBR Green/ROX qPCR Master Mix kit (Thermo Fisher Scientific, Waltham, MA, USA). The qRT-PCR reaction consisted of 9 μL of Maxima SYBR Green/ROX qPCR Master Mix (2×) (Thermo Fisher Scientific, USA), 5 μL of cDNA (25 ng/μL), 1 μL of forward and reverse primer (0.4 μM), and 4 μL of water for a final reaction volume of 20 μL in each tube. The final qRT-PCR reactions were run by triplicate from two biological replicates. The qRT-PCR assay consisted of a two-step protocol with initial activation of polymerase step of 95 °C for 10 min, followed by 40 cycles of 95 °C for 20 s and 35 s of melting at 56 °C for *SOD* and 58 °C for *PPO*. The fluorescence was detected at the melting temperature during each cycle and the expression levels were normalized using the *UBC* reference gene according to that reported by Berumen-Varela et al. [48] in soursop fruits. The relative expression level for each gene was calculated by the formula 2^−∆∆Ct^ as reported by [49]. 

#### 4.5.5. Statistical Analysis

A complete 3 × 2 factorial experimental design (days of storage, inoculation) was used. A total of six treatments were evaluated. Data were analyzed using two-way analysis of variance (ANOVA) with a significance level of 5% using RStudio. The interaction effect between days of storage and inoculation was also calculated. Tukey’s multiple comparison post hoc test was carried out on all the data (*p <* 0.05) with the agricolae package. Furthermore, pairwise Pearson correlation analysis was performed to evaluate the relationship between the parameters evaluated using the corrplot and Hmisc packages. Moreover, all the variables evaluated were subjected to principal component analysis (PCA) to analyze their importance in the fruit–pathogen interaction with the FactoExtra and FactoMineR packages.

## 5. Conclusions

Here, we show that *N. haematococca* induced the antioxidative system in soursop fruits during postharvest storage. High response was observed on day one, in the total phenols (variable that contributes the most to the fruit–pathogen interaction), on day three, in the relative gene expression of *PPO* and *SOD*, and on day 5, the *PPO* enzyme activity. Furthermore, this is the first work that has demonstrated the differential response in the antioxidative system during the interaction of soursop–*N. haematococca* at postharvest storage.

## Figures and Tables

**Table 1:** Sequences of primers.

Gene	Primer sequence 5′–3′	Size (bp)
*PPO*	Fw: AAGCCAGCATCCGAAGAGAG	150
	Rv: GTCGTAGCTCAGCTGCTTCA	
*SOD*	Fw: GGCCAAACTCCATCATTG Rv: GCATTTCCAGTGGTCTTG	97
*UBC*	Fw: AACCTCTATCCAGTCTCTCCTC	128
	Rv: TGAGATAGTGGAGCAGAGCT	

Fw and Rv represent forward and reverse primers, respectively.

## Data Availability

Data is contained within the article.

## Supplementary Materials

The following are available online at https://www.mdpi.com/article/10.3390/plants10071432/s1, Table S1. Two-way ANOVA of all the variables analyzed, Table S2. Eigenvalue, variance percent and cumulative variance percent of the principal component analysis, Table S3. Correlation coefficients values (r), Table S4. Correlation coefficients values (*p*).
