# Supplementary material for: Differential Responses of Antioxidative System during the Interaction of Soursop Fruits (Annona muricata L.) and Nectria haematococca at Postharvest Storage
Source: Plants (Basel). 2021 Jul 14;10(7):1432. doi: 10.3390/plants10071432 (PMC8309373; doi:10.3390/plants10071432)
Supplement: Supplementary file 1 [file plants-10-01432-s001.zip › plants-1282954-supplementary.pdf]

**Table S1. Two-way ANOVA of all the variables analyzed.**

| Total Phenols | Df | Sum Sq | Mean Sq | F value | Pr(>F) |
|---|---|---|---|---|---|
| **Day** | 2 | 38619 | 19310 | 49.531 | 9.31e-16 *** |
| **Inoculation** | 1 | 192 | 192 | 0.492 | 0.48459 |
| **Day:Inoculation** | 2 | 5496 | 2748 | 7.048 | 0.00136 ** |
| **Residuals** | 102 | 39765 | 390 | | |

| DPPH | Df | Sum Sq | Mean Sq | F value | Pr(>F) |
|---|---|---|---|---|---|
| **Day** | 2 | 58281 | 29141 | 15.372 | 1.46e-06 *** |
| **Inoculation** | 1 | 723 | 723 | 0.381 | 0.53825 |
| **Day:Inoculation** | 2 | 25663 | 12832 | 6.769 | 0.00174 ** |
| **Residuals** | 102 | 193358 | 1896 | | |

| ABTS | Df | Sum Sq | Mean Sq | F value | Pr(>F) |
|---|---|---|---|---|---|
| **Day** | 2 | 82845 | 41422 | 5.033 | 0.00823 ** |
| **Inoculation** | 1 | 29282 | 29282 | 3.558 | 0.06210 . |
| **Day:Inoculation** | 2 | 43872 | 21936 | 2.665 | 0.07441 . |
| **Residuals** | 102 | 839423 | 8230 | | |

| FRAP | Df | Sum Sq | Mean Sq | F value | Pr(>F) |
|---|---|---|---|---|---|
| **Day** | 2 | 739.0 | 369.5 | 25.756 | 8.82e-10 *** |
| **Inoculation** | 1 | 56.9 | 56.9 | 3.967 | 0.04909 * |
| **Day:Inoculation** | 2 | 200.9 | 100.4 | 7.000 | 0.00142 ** |
| **Residuals** | 102 | 1463.4 | 14.3 | | |

| PPO activity | Df | Sum Sq | Mean Sq | F value | Pr(>F) |
|---|---|---|---|---|---|
| **Day** | 2 | 24434 | 12217 | 18.247 | 0.00023 *** |
| **Inoculation** | 1 | 29498 | 29498 | 44.056 | 2.4e-05 *** |
| **Day:Inoculation** | 2 | 13022 | 6511 | 9.725 | 0.00309 ** |
| **Residuals** | 12 | 8035 | 670 | | |

| SOD activity | Df | Sum Sq | Mean Sq | F value | Pr(>F) |
|---|---|---|---|---|---|
| **Day** | 2 | 1428.2 | 714.1 | 22.977 | 7.88e-05 *** |
| **Inoculation** | 1 | 2.7 | 2.7 | 0.087 | 0.773 |
| **Day:Inoculation** | 2 | 125.2 | 62.6 | 2.014 | 0.176 |
| **Residuals** | 12 | 372.9 | 31.1 | | |

| SOD expression | Df | Sum Sq | Mean Sq | F value | Pr(>F) |
|---|---|---|---|---|---|
| **Day** | 2 | 203.22 | 101.61 | 5.077 | 0.0253 * |
| **Inoculation** | 1 | 63.66 | 63.66 | 3.181 | 0.0998 . |
| **Day:Inoculation** | 2 | 203.22 | 101.61 | 5.077 | 0.0253 * |
| **Residuals** | 12 | 240.14 | 20.01 | | |

| PPO expression | Df | Sum Sq | Mean Sq | F value | Pr(>F) |
|---|---|---|---|---|---|
| **Day** | 2 | 29.202 | 14.601 | 13.730 | 0.000791 *** |
| **Inoculation** | 1 | 6.469 | 6.469 | 6.082 | 0.029697 * |
| **Day:Inoculation** | 2 | 29.202 | 14.601 | 13.730 | 0.000791 *** |
| **Residuals** | 12 | 12.762 | 1.063 | | |

Significant codes: 0 '***' 0.001 '**' 0.01 '*' 0.05 '.' 0.1 ' ' 1

**Table S2. Eigenvalue, variance percent and cumulative variance percent of the principal component analysis**

| | eigenvalue | variance.percent | cumulative.variance.percent |
|---|---|---|---|
| **PC1** | 3.525640 | 4.407050e+01 | 44.07050 |
| **PC2** | 2.179665 | 2.724582e+01 | 71.31631 |
| **PC3** | 1.935024 | 2.418780e+01 | 95.50412 |
| **PC4** | 0.2553629 | 3.192036e+00 | 98.69615 |
| **PC5** | 0.1043076 | 1.303845e+00 | 100.00000 |
| **PC6** | 7.507267e-32 | 9.384084e-31 | 100.00000 |

**Table S3. Correlation coefficients values (r)**

| | Phenols | DPPH | ABTS | FRAP | PPO_act | SOD_act | PPO_exp | SOD_exp |
|---|---|---|---|---|---|---|---|---|
| **Phenols** | 1.00000 | -0.931751 | 0.44702 | -0.71443 | -0.76986 | 0.962392 | -0.292298 | -0.40981 |
| **DPPH** | -0.93175 | 1.00000 | -0.223712 | 0.8578296 | 0.60740 | -0.989137 | 0.014517 | 0.11820 |
| **ABTS** | 0.44702 | -0.223712 | 1.000000 | 0.29094 | -0.90866 | 0.354777 | -0.068764 | -0.19498 |
| **FRAP** | -0.71443 | 0.857829 | 0.290942 | 1.00000 | 0.133749 | -0.785045 | 0.115113 | 0.14903 |
| **PPO_act** | -0.76986 | 0.607400 | -0.908663 | 0.13374 | 1.000000 | -0.711735 | 0.104409 | 0.250250 |
| **SOD_act** | 0.96239 | -0.989137 | 0.354777 | -0.78504 | -0.71173 | 1.00000 | -0.058332 | -0.17448 |
| **PPO_exp** | -0.29229 | 0.014517 | -0.068764 | 0.11511 | 0.1044090 | -0.058332 | 1.00000 | 0.98882 |
| **SOD_exp** | -0.40981 | 0.118205 | -0.194988 | 0.14903 | 0.25025 | -0.174482 | 0.988828 | 1.00000 |

**Table S4. Correlation coefficients values (p)**

| | Phenols | DPPH | ABTS | FRAP | PPO_act | SOD_act | PPO_exp | SOD_exp |
|---|---|---|---|---|---|---|---|---|
| **Phenols** | NA | 7.5461e-04 | 0.2668018 | 0.0464602 | 0.0254524 | 1.2925e-04 | 4.8235e-01 | 3.1329e-01 |
| **DPPH** | 0.0007546 | NA | 0.5943237 | 0.0064397 | 0.1102350 | 3.1784e-06 | 9.7278e-01 | 7.8042e-01 |
| **ABTS** | 0.2668018 | 5.9432e-01 | NA | 0.4844859 | 0.0017768 | 3.8850e-01 | 8.7147e-01 | 6.4355e-01 |
| **FRAP** | 0.0464602 | 6.4397e-03 | 0.4844859 | NA | 0.7521941 | 2.0999e-02 | 7.8606e-01 | 7.2466e-01 |
| **PPO_act** | 0.0254524 | 1.1023e-01 | 0.0017768 | 0.7521941 | NA | 4.7684e-02 | 8.0565e-01 | 5.5000e-01 |
| **SOD_act** | 0.0001292 | 3.1784e-06 | 0.3885029 | 0.0209993 | 0.0476840 | NA | 8.9087e-01 | 6.7942e-01 |
| **PPO_exp** | 0.4823572 | 9.7278e-01 | 0.8714718 | 0.7860621 | 0.8056511 | 8.9087e-01 | NA | 3.4563e-06 |
| **SOD_exp** | 0.3132948 | 7.8042e-01 | 0.6435574 | 0.7246658 | 0.5500031 | 6.7942e-01 | 3.4563e-06 | NA |
